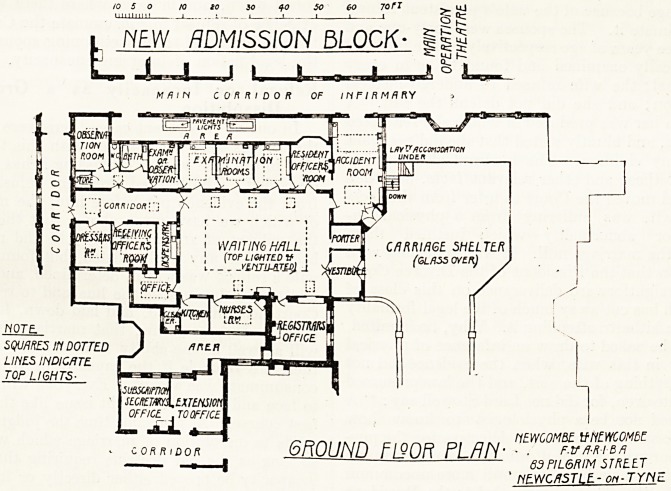# New Admission Block at Royal Victoria Infirmary, Newcastle

**Published:** 1913-10-04

**Authors:** 


### THE ADMISSIONS TO A VOLUNTARY HOSPITAL.

### The New Admission Block at the Royal Victoria Infirmary, Newcastle-upon-Tyne.

The admission of patients to a modern hospital is a task of considerable difficulty. The number of applicants is always in excess of the accommodation available, and it is not an easy matter to decide which out of a number of cases should be admitted first. Nor is the task, difficult enough from a professional point of view, rendered any easier by the great variety in the social conditions of the applicants. One has a wife and family, another is unmarried; is the dependent family to outweigh the, possibly, greater urgency of the complaint of the unmarried? and so on *ad infinitum*.

### Unmerited Hospital Criticism.

Although these difficulties have been, and still are being, overcome with a great degree of success, yet hospitals are subjected to much unmerited criticism, and the reason for this is not that there is any conscious unfairness in the methods employed, but because there is in any one hospital a lack of uniformity in these methods, and this lack of uniformity is bound to exist where there is more than one admitting authority.

Ten or a dozen years ago the system, or rather the lack of system, worked well enough: each honorary physician and each honorary surgeon was an admitting authority, and as the pressure of beds was less than it is nowadays there was less difficulty in coming to a decision; but as the number of applicants for admission increased, and as, in many hospitals, the number of admitting authorities was increased also, for beds were given to assistant physicians, assistant surgeons, and so on, the system gradually broke down, and complaints increased to such an extent that committees of management were compelled to take the whole matter into consideration and endeavour to devise some scheme for the admission of patients which would give general satisfaction.

### The Newcastle Plan.

At the Royal Victoria Infirmary, Newcastle-upon-Tyne, a new admission block has been built. This block is under the charge of a resident medical officer of greater experience tnan the average hospital resident physician or surgeon. Through his hands all admissions are made, both to the in-patient and out-patient departments. He is responsible for the keeping of the waiting list and for the admission of patients from this waiting list. Adequate assistance—professional, nursing, and clerical—has been given him to carry out his work. At the same time the social circumstances of all applicants are inquired into so that they may become a definite factor in deciding for or against admission. In the admission block arrangements have been made for the registration of patients and for the investigation of the various cases. At the same time the committee fully realise that any system to be successful must have a certain amount of elasticity, and they have conceded to members of the honorary staff the right to admit a certain number of request cases each month to the beds under their charge.

With regard to the site of the admission block, the committee had not a great deal of choice. After careful consideration they came to the conclusion that, in spite of certain disadvantages, the central site was the best. They cleared the area in which stood the old accident room and its adjoining rooms and built the new admission block in its place. The accompanying plan makes the arrangements of the rooms clear. The walls of the waiting hall, the accident room, the examination rooms, etc., are tiled, and the floors are of terrazzo. The hall and most of the rooms have top as well as side lights. Lavatory accommodation has been provided for the patients in the area at the foot of the steps beside the accident room. The rooms have been in occupation for three months and, we are informed, have been found most satisfactory. The light is good and the ventilation excellent.

## Figures and Tables

**Figure f1:**